# Development and In Vivo Evaluation of a Novel Bioabsorbable Polylactic Acid Middle Ear Ventilation Tube

**DOI:** 10.3390/jfb17010025

**Published:** 2025-12-30

**Authors:** Ying-Chang Lu, Chi-Chieh Chang, Ping-Tun Teng, Chien-Hsing Wu, Hsuan-Hsuan Wu, Chiung-Ju Lin, Tien-Chen Liu, Yen-Hui Chan, Chen-Chi Wu

**Affiliations:** 1 Department of Otolaryngology-Head and Neck Surgery, National Taiwan University Hospital, Taipei 100, Taiwan; lyc16889@ntu.edu.tw (Y.-C.L.); r07442023@ntu.edu.tw (C.-C.C.); liuent@ntu.edu.tw (T.-C.L.); 2 SG Biomedical Co., Ltd., Hsinchu County 303, Taiwan; david.teng@sghlds.com (P.-T.T.); sam.wu@sghlds.com (C.-H.W.); hsuwu@ucdavis.edu (H.-H.W.); kyle.lin@sghlds.com (C.-J.L.); 3 Department of Pediatrics, National Taiwan University Hospital, Taipei 100, Taiwan; 4 Department of Otolaryngology Head and Neck Surgery, National Taiwan University Hospital Hsin-Chu Branch, Hsinchu County 302, Taiwan

**Keywords:** middle ear ventilation tube, polylactic acid, absorbable, biocompatibility

## Abstract

Background: Otitis media with effusion (OME) is a widespread condition that causes hearing impairment, particularly in pediatric populations. Existing non-absorbable tubes often require elective or unplanned removal surgery. Bioabsorbable polylactic acid (PLA) offers a promising alternative due to its inherent biocompatibility and tunable degradation characteristics. In this study, we designed, fabricated, and comprehensively evaluated a novel PLA middle-ear ventilation tube. Methods: Bioabsorbable PLA tubes were designed and fabricated based on commercial models. In vitro biocompatibility was assessed according to ISO 10993 guidelines. A guinea pig model was used to perform in vivo evaluations, including otoscopic examinations, auditory brainstem response (ABR) measurements, micro-computed tomography (micro-CT) imaging, and histological analyses. Results: The PLA tubes were successfully designed and fabricated, exhibiting dimensions comparable to those of commercially available products. In vitro testing confirmed their biocompatibility. In vivo observations revealed that the PLA segments remained stable, with no significant inflammation detected. ABR measurements revealed no adverse impacts on hearing function. Micro-CT imaging confirmed tube integrity and indicated initial signs of degradation over a 9-month period, as evidenced by radiographic morphology. Histological analyses indicated a favorable tissue response with minimal foreign body reaction. Conclusions: The developed PLA middle-ear ventilation tube represents a highly promising alternative to conventional non-absorbable tubes. It demonstrates excellent biocompatibility, preserves auditory function, and exhibits a controlled degradation profile. This preclinical study provides strong support for further investigation and subsequent clinical trials to validate its safety and efficacy in human patients.

## 1. Introduction

Otitis media with effusion (OME) is a prevalent inflammatory condition characterized by accumulation of fluid in the middle-ear space without acute signs of infection. OME is a leading cause of hearing impairment in children, with an estimated prevalence of up to 10% in pediatric populations [1,2]. The eustachian tube, connecting the middle ear to the nasopharynx, is crucial for maintaining middle-ear pressure equilibrium and drainage. Dysfunction of this tube, often due to immature development in children or conditions such as allergic rhinitis, sinusitis, or nasopharyngeal carcinoma in adults, can lead to OME [2]. Prolonged untreated OME (e.g., 2–3 months) can result in significant and potentially irreversible hearing loss, impacting speech development, learning, and overall quality of life [1].

For OME that does not respond to medical treatment, middle-ear ventilation tubes are a common and effective surgical intervention [1,3]. The procedure involves making a small incision in the tympanic membrane (myringotomy) [4,5,6,7,8] to drain fluid, followed by tube placement to maintain aeration and equalize pressure [9]. Unlike simple myringotomies, which heal rapidly, the tube prevents premature closure, ensuring prolonged ventilation (ideally 6–24 months) and fluid drainage while eustachian tube function recovers [1,9]. Over one million tympanostomy tube insertions are performed annually in the U.S., making it one of the most common pediatric surgical procedures [10]. The global clinical demand for these tubes underscores the need for improved solutions.

Current non-absorbable middle-ear ventilation tubes, typically made from Teflon or silicone, are associated with significant limitations [11], including failure to spontaneously extrude in up to 40% of cases [12,13]. Retained tubes can trigger foreign body reactions, leading to granulation tissue, inflammation, and tympanosclerosis [14]. Additionally, a second surgical procedure is often required for tube removal, raising healthcare costs and patient risks and exposing pediatric patients to additional risks under general anesthesia [14,15]. Patients with eustachian tube dysfunction may develop retraction pockets from atrophic membranes, which are a precursor to cholesteatoma; perforations often require surgical repair. Rare but serious complications, such as opportunistic mycobacterial infections [16,17], have also been reported [18,19]. These issues underscore the urgent clinical need for a novel middle-ear ventilation tube that degrades predictably, thus eliminating the need for a second intervention.

In an effort to overcome the limitations of non-absorbable tubes, researchers have explored bioabsorbable alternatives. Previous attempts using materials such as poly-bis(ethylalanate)phosphazene, hyaluronic acid, and gelatin have shown promise in terms of biocompatibility and ventilation function. However, these materials often exhibited overly rapid absorption rates or caused inflammation, which limited their clinical applicability [20,21]. Polylactic acid (PLA) is a well-established bioabsorbable material widely used in surgical sutures, bone fixation [22,23,24], and drug delivery systems, owing to its FDA approval, biocompatibility, and biodegradability [25,26]. Derived from renewable plant sources [25], PLA degrades into lactic acid, water, and carbon dioxide [27,28], which are naturally metabolized and safely cleared from the body. These properties, along with its ISO 10993-certified biocompatibility, support its safety for clinical use. In the context of middle-ear ventilation, PLA was specifically selected over other bioabsorbable alternatives like poly(lactic-co-glycolic acid) (PLGA) or poly-bis(ethylalanate)phosphazene (PBE) primarily due to its superior hydrolytic stability. While PLGA and PBE formulations often degrade rapidly—sometimes in as little as 18 days [20,29]—PLA offers prolonged structural integrity [30] specifically designed to match the ideal 1–2 year clinical ventilation period [3]. Furthermore, PLA’s thermoplastic nature enables precise injection molding for scalable, high-quality manufacturing, and its inherent bacteriostatic properties provide an additional strategic advantage in the infection-prone middle-ear environment.

PLA’s mildly acidic degradation environment may offer bacteriostatic properties, and it can be engineered with antimicrobial coatings or for drug loading. Given the advantages of PLA and the significant unmet clinical need, the objective of this study was to design, manufacture, and evaluate a novel bioabsorbable PLA middle-ear ventilation tube. We aimed to develop an optimized PLA tube with controlled degradation kinetics to effectively ventilate the middle ear.

## 2. Materials and Methods

### 2.1. Design and Fabrication of PLA Middle Ear Ventilation Tubes

**Figure 1:**
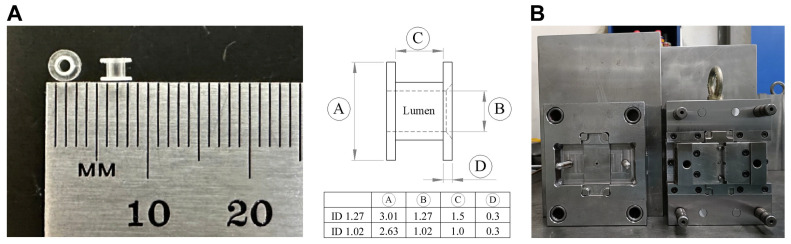
Design of the bioabsorbable middle ear ventilation tube. (**A**) Image of the custom-designed PLA tube alongside a ruler, showing its dimensions. The accompanying table compares the outer diameter (OD) and inner diameter (ID) of the PLA material used in this study (3.01 mm OD, 1.27 mm ID, and 2.63 mm OD, 1.02 mm ID) with standard Sheehy-Type Collar Buttons (3.0 mm OD, 1.27 mm ID) and Reuter Bobbins (2.7 mm OD, 1.02 mm ID). (**B**) The medical-grade stainless steel mold developed for the consistent production of the PLA tubes via injection molding.

The middle-ear ventilation tube was designed to replicate the form and function of existing commercial products. As depicted in Figure 1, the tube features a dumbbell shape with a central lumen to facilitate exudate flow and pressure equalization. PLA was selected as the raw material because of its biodegradable and thermoplastic properties. A medical-grade stainless-steel mold was developed to facilitate injection molding, utilizing a Sumitomo injection-molding machine (se100d, Sumitomo Heavy Industries, Tokyo, Japan). Injection molding was chosen for its suitability for mass production and ability to ensure consistent product quality, aspects that are crucial for future product certification and commercialization.

### 2.2. Mechanical and Biocompatibility Assessments

The product was packaged using Tyvek^®^ pouch (DuPont, Wilmington, DE, USA), and gamma irradiation was chosen as the sterilization method. A dose audit subsequently confirmed the sterility of the packaged products, with an applied dose ranging from 10 to 15 kGy. A sterilization dose of 15 kGy was selected and validated using ISO 11137-2 [31] Method VD_max_^15^ to achieve a sterility assurance level (SAL) of 10^−6^ while minimizing radiation-induced chain scission of the PLA material. In terms of biocompatibility, the ventilation tube was categorized as Category C according to ISO 10993-1 [32] standards. Both cytotoxicity and sensitization tests were completed and yielded acceptable results. The mechanical strength of the PLA tube was evaluated using a pressure test in which axial and radial forces were applied to the product. The force required for tube collapse or displacement was recorded to compare strength with that of commercial models.

To assess the long-term degradation profile, PLA tubes were immersed in phosphate-buffered saline (PBS) and incubated at 37 °C. At predetermined time points (e.g., up to 18 months), samples were removed, dried, and analyzed. Weight loss was determined by measuring the remaining mass, and the residual mechanical strength was reevaluated using the same axial and radial pressure tests. Overall, the product fulfills the sterility, mechanical, and biocompatibility requirements, from raw material selection through manufacturing, sterilization, and packaging, making it suitable for subsequent animal studies.

### 2.3. Animal Model Development and In Vivo Safety Evaluation

Guinea pigs were housed at the Laboratory Animal Center of the College of Medicine, National Taiwan University. All experimental procedures were conducted in accordance with institutional animal welfare guidelines and approved by the Institutional Animal Care and Use Committee (IACUC) of the National Taiwan University College of Medicine (Approval No. 20240003).

**Figure 2:**
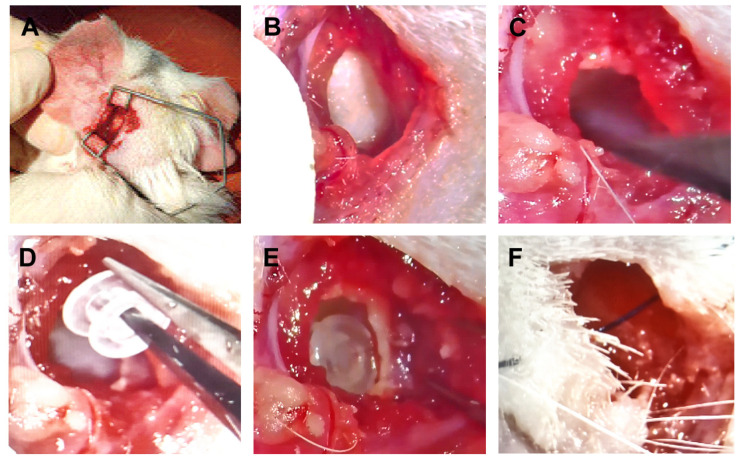
Surgical implantation of PLA tubes in the guinea pig model. (**A**) Preparation for surgery, with the ear secured. (**B**) A closer view of the surgical site, revealing the incision. (**C**) Use of a probe within the incision. (**D**) Insertion of the PLA material using forceps. (**E**) PLA tube in place within the surgical site. (**F**) The ear after the procedure, with the incision closed.

A comprehensive in vivo model was established using male guinea pigs weighing 200–250 g for the evaluation of middle-ear implantation. All animals were acclimatized in the animal facility for 7 days prior to the initiation of experiments. Experimental units were randomly allocated to groups. Surgical techniques were developed for placing materials into the middle-ear cavity. Anesthesia was induced using 3% inhalational isoflurane and maintained at a level sufficient to keep the animals sedated during the procedure and auditory brainstem response (ABR) testing. The surgical site was prepared by shaving the fur behind the left ear to expose the skin (Figure 2). The contralateral ear served as a surgical control (Mock), in which the mastoid portion of the ventral tympanic bulla was opened without implanting a ventilation tube. Under microscopic magnification (Zeiss, Oberkochen, Germany), a 1–1.5 cm post-auricular skin incision was made using a scalpel. Blunt dissection was performed through the subcutaneous fat layer, which varied in thickness among animals. The bulla was accessed by gently rotating a scalpel to pierce the posterior–superior aspect of the ridge of the tympanic bulla. A small fenestration was created in the mastoid portion of the ventral bulla, and the ventilation tube was inserted into the middle-ear cavity using forceps (Figure 2).

### 2.4. Audiological Evaluations

ABR thresholds were assessed in guinea pigs using an evoked potential detection system (Smart EP 3.90, Intelligent Hearing Systems, Miami, FL, USA), as previously described [33]. Auditory stimuli, including click sounds and tone bursts at 8, 16, and 32 kHz, were presented at varying intensities from 10 to 90 dB SPL. ABR responses were recorded using subcutaneous needle electrodes inserted ventrolaterally near the ears. Waveforms and thresholds were measured and monitored throughout the observation period, with comparisons made to baseline levels to evaluate changes in auditory function.

### 2.5. Imaging and Histological Studies

Otoscopic examination was performed to assess the general condition of the tympanic membrane and external auditory canal prior to further imaging. Micro-computed tomography (micro-CT) imaging was employed to perform non-destructive, in vivo visualization of the implanted tube’s position, structural integrity, and any early signs of degradation [21]. Representative CT scans were obtained periodically, up to 9 months post-implantation. Gross anatomical evaluation was conducted during necropsy to assess macroscopic tissue responses and implant site morphology. For histopathological analysis, cochleae were harvested at 18 months postoperatively. Samples were fixed in 10% paraformaldehyde for 48 h, decalcified, embedded in paraffin, sectioned into 8 μm slices, and stained with hematoxylin and eosin (H&E).

### 2.6. Statistical Analysis

All statistical analyses were conducted using GraphPad Prism version 9 (GraphPad Software Inc., San Diego, CA, USA). For datasets involving two independent variables, two-way analysis of variance (ANOVA) was applied, followed by Dunnett’s post hoc multiple comparisons test to evaluate group differences relative to the designated control condition.

## 3. Results

### 3.1. Design and Fabrication of PLA Middle-Ear Ventilation Tube

Two bioabsorbable middle ear ventilation tube prototypes were successfully fabricated. Figure 1A presents the final design specifications, featuring a dumbbell shape with dimensions (e.g., 3.01 mm OD by 1.27 mm ID) comparable to those of commercial standards, including Sheehy-type collar buttons and Medtronic models. The injection molding process yielded PLA tubes with consistent structural quality and high dimensional accuracy (Figure 1B), demonstrating the feasibility of the production process.

### 3.2. Mechanical Strength and In Vitro Degradation

**Figure 3:**
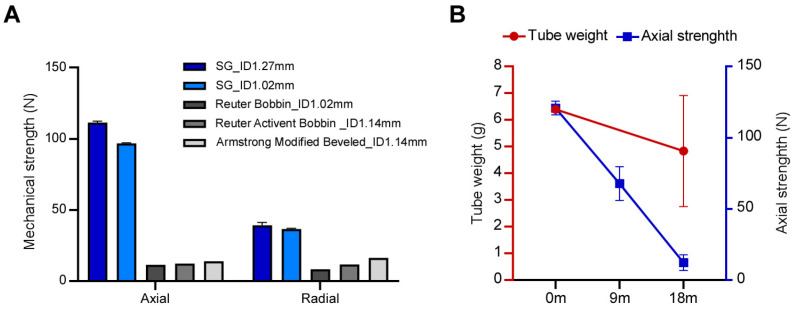
Mechanical properties of the bioabsorbable middle ear ventilation tube. (**A**) Pressure test demonstrating the superior mechanical strength of the PLA tube (*n* = 3 for axial; *n* = 4 for radial) compared to various commercially available non-absorbable tubes (*n* = 1 per group) when subjected to axial and radial forces. (**B**) The in vitro degradation profile of the bioabsorbable middle ear ventilation tube shows the percentage of weight and mechanical strength retained over 0 to 18 months (*n* = 7 for 0 m; *n* = 2 for 9 m; *n* = 3 for 18 m) in PBS at 37 °C. The results indicate that the decline in mechanical strength precedes measurable weight loss, consistent with bulk erosion. All data are presented as mean ± standard deviation (SD).

As illustrated in Figure 3A, the PLA tube exhibited superior mechanical strength compared to commercial benchmarks under axial and radial loading. Over the 18-month observation period (Figure 3B), mass retention decreased gradually from an initial 7.05 mg to 4.83 mg (68.55% retention). In contrast, the initial load-bearing capacity of 111.34 N dropped to 67.72 N (60.82% retention) at 9 months and further to 12.18 N (10.95% retention) by 18 months. Mechanical strength exhibited a significantly steeper decline, which is consistent with the mechanism of PLA bulk erosion, where hydrolysis-induced chain scission leads to a significant reduction in mechanical strength and molecular weight prior to measurable mass loss. We acknowledge that molecular weight analysis (e.g., GPC) was not performed to quantify this chain scission, but the observed degradation pattern aligns with established PLA behavior. Collectively, the degradation and pressure data suggest that the tube retains its functionality throughout the clinically relevant indwelling period and then gradually resorbs over extended periods, thus achieving controlled, sequential degradation.

### 3.3. Biocompatibility

The PLA middle-ear ventilation tube passed preliminary evaluations, including injection molding, packaging, and sterilization. The results of the Limulus amebocyte lysate (LAL) assay indicated an endotoxin concentration of <0.5 EU/device. Regarding biocompatibility, the tube yielded acceptable results regarding the cytotoxicity and sensitization tests. This comprehensive success across sterility, mechanical, and biocompatibility requirements confirms the product’s suitability for subsequent animal studies.

### 3.4. Animal Model Development

A comprehensive Guinea pig animal model was established for in vivo evaluation. Figure 2 illustrates the successful development of surgical techniques for implanting materials into the middle-ear cavity.

### 3.5. Audiological Results

**Figure 4:**
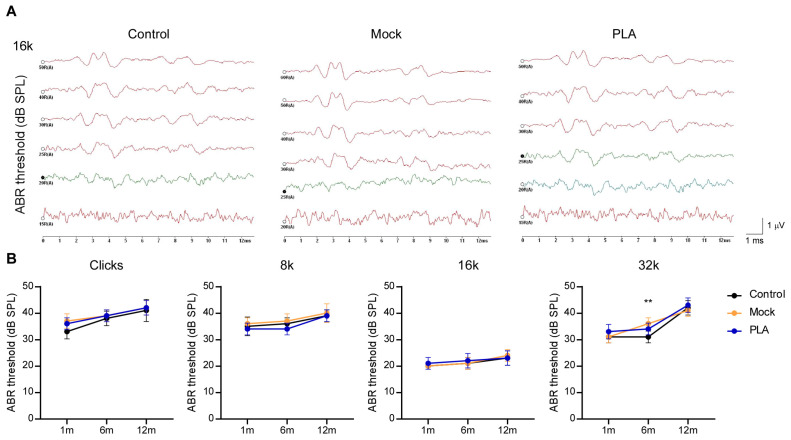
Longitudinal hearing results after PLA tube implantation. (**A**) Representative ABR waveforms at decreasing sound pressure levels (dB SPL) for the control, mock, and PLA groups, respectively, using a 16 kHz stimulus. Positive waveforms were detected at 20 dB SPL in both the control and mock groups, and at 25 dB SPL in the PLA group. (**B**) Comparison of ABR thresholds in dB SPL for the control, mock, and PLA groups (*n* = 5 per group) over 1, 6, and 12 months for each frequency, demonstrating no difference between the three groups. All data are presented as mean ± standard deviation (SD). *p*-values were determined via Dunnett’s two-way ANOVA. **: *p* < 0.01 for Control vs. Mock at 6 m of 32 k stimulus.

In this preliminary safety screening, the guinea pigs in the control (pre-surgery), mock (surgical control), and PLA groups (*n* = 5 per group) maintained normal hearing thresholds at 1, 6, and 12 months post-operation, as shown in Figure 4. While the sample size limited quantitative statistical power, the consistent absence of significant threshold shifts suggests that PLA tube implantation did not have any gross adverse impacts on auditory function. ABR waveforms and thresholds remained stable and comparable to baseline values, suggesting that PLA tube implantation preserved normal middle-ear function, which is essential for efficient sound conduction during the entire observation period.

### 3.6. Imaging and Histology Results

**Figure 5:**
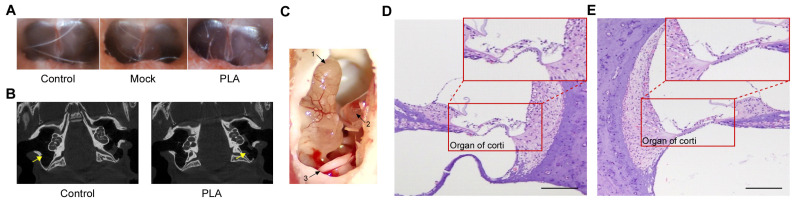
In vivo evaluation of PLA tubes via imaging and histology. (**A**) Representative otoscopic images of the tympanic membrane at 12 months after implantation of the PLA tube, demonstrating their stable position and absence of visible inflammation or adverse reactions. (**B**) Micro-CT images taken up to 9 months post-implantation, illustrating the sustained position and structural integrity of the implanted PLA tubes within the middle ear cavity, along with early signs of material degradation. Arrows indicate the PLA tube in the implanted group and the corresponding region in the control group. (**C**) Gross anatomical assessment performed during necropsy 12 months after implantation reveals an intact middle ear and tympanic membrane, with minimal foreign body response at the implant site. Arrow 1: cochlea, Arrow 2: tympanic membrane, Arrow 3: PLA tube. (**D**,**E**): Cochlear histology harvested 18 months after implantation shows that normal cochlear hair cells were preserved in both the mock and PLA groups. (bar = 200 µm).

Otoscopic examinations performed at 1, 6, and 12 months post-implantation revealed no visible inflammation or adverse reactions in the tympanic membrane or middle ear at any point for up to 12 months (Figure 5A). Specifically, no evidence of hemotympanum or tympanic membrane perforation was observed in any of the groups, and the tympanic membranes in all groups remained normal throughout the study. These observations indicate the in vivo biocompatibility and mechanical stability of the PLA material.

Representative micro-CT images (Figure 5B), obtained up to 9 months post-implantation, confirmed the preservation of tube morphology within the middle-ear cavity. Initial signs of biodegradation were observed, as evidenced by radiographic morphology. Gross anatomical assessment during a necropsy further corroborated these findings, revealing an intact middle ear and tympanic membrane and minimal foreign body response at the implant site (Figure 5C). Cochlear histology at 18 months showed normal cochlear hair cells in both the mock (Figure 5D) and PLA (Figure 5E) groups. The preservation of normal cochlear hair cells in both groups at 18 months indicates that neither the PLA tube nor the surgical procedure itself resulted in a significant inflammatory response or hair cell loss.

## 4. Discussion

### 4.1. Summary of Key Findings

In this study, we successfully designed, fabricated, and preclinically evaluated a novel bioabsorbable middle-ear ventilation tube utilizing PLA as the primary material. Our results demonstrate the successful development of the prototype with dimensions comparable to commercially available non-absorbable tubes, confirming the feasibility of the manufacturing process using optimized injection-molding techniques. Importantly, in vitro mechanical and biocompatibility assessments, including pressure, cytotoxicity, and sensitization tests, confirmed that the material was safe based on its safety profile. The in vivo evaluation using a guinea pig model further validated the safety and biocompatibility of the PLA material, showing no significant inflammation or adverse reactions. Crucially, ABR measurements indicated that the implanted PLA tubes did not adversely affect hearing function. Advanced micro-CT imaging provided in vivo evidence of the tube’s sustained position and initial degradation over 9 months, while histological analyses corroborated the favorable tissue response and absence of significant foreign-body reactions. These findings collectively establish our PLA middle-ear ventilation tube as a promising solution with appropriate biocompatibility and an observable degradation profile.

### 4.2. Advantages over Existing Products

A key innovation of this study is the development of a bioabsorbable PLA middle-ear ventilation tube, presenting a potential alternative to traditional non-absorbable devices (e.g., Teflon, silicone, and stainless steel). Current devices often necessitate secondary removal surgery due to complications like extrusion failure, granulation tissue, tympanosclerosis, and persistent tympanic membrane perforation [21,34]. In contrast, our PLA tube is designed to be absorbed within the middle ear over a clinically relevant timeframe (1–2 years). This design is intended to mitigate the need for additional interventions, thereby potentially reducing patient risks, healthcare costs, and long-term tympanic membrane complications.

Several types of biomaterials have been applied to develop absorbable middle-ear ventilation tubes (Table 1). D’Eredità and Marsh (2005) found that poly-bis(ethylalanate)phosphazene (P-PHOS) tubes in guinea pigs showed excellent tympanic membrane healing with no infection or inflammatory reaction, and their disintegration rate could be controlled by varying the polymer formulation [20]. In a guinea pig model, Massey et al. observed that PLA tubes remained in the tympanic membrane longer (for an average of 63.2 vs. 19.3 days) than 50/50 PLA/PLGA tubes (for an average of 18.8 vs. 8.1 days), with both demonstrating good biocompatibility and complete tympanic membrane healing [29]. Ludwick et al. showed that PLA tubes possessed bacteriostatic properties against *Pseudomonas aeruginosa* and *Staphylococcus aureus* in vitro [34], suggesting a potential reduction in post-placement otorrhea. Park et al. further explored polyester tubes in chinchillas, noting that while PLGA tubes showed more inflammation, silver-coated PLGA tubes led to significantly less fibrosis and lower neutrophil counts [21], indicating an anti-inflammatory effect. Skovlund et al. conducted a human feasibility study, demonstrating that a novel bioabsorbable gelatin ventilation tube provided intermediate-term middle-ear ventilation for an average of 12 weeks, supporting its clinical applicability [35].

Compared to previous studies, our research on a novel bioabsorbable PLA middle-ear ventilation tube in guinea pigs demonstrates notable strengths. Earlier P-PHOS tubes degraded within 30 to 60 days, and PLA/PLGA tubes were resorbed within 18.8 to 63.2 days in guinea pigs and within 18.9 days for PLGA and over 30 days for PLA in chinchillas, but our PLA tubes exhibited sustained structural integrity, with initial signs of degradation appearing after nine months (over 270 days). This prolonged, controlled degradation profile in vivo is specifically designed to match the ideal 1–2-year clinical ventilation period and represents a considerable advantage over prior materials that often degrade too rapidly. Furthermore, our PLA tubes demonstrated excellent biocompatibility. We did not observe any significant inflammation, foreign-body reactions, or negative impacts on hearing function, as measured via ABR. This finding is consistent with previous PLA findings. Unlike some cross-linked hydrogel tubes, which caused high levels of inflammation and enlarged the myringotomy site, our PLA design avoids such adverse responses. Successful fabrication via injection molding ensures consistent quality and suitability for mass production. This comprehensive preclinical evaluation showing favorable biocompatibility, maintained hearing, and a significantly extended and controlled degradation time positions our PLA ventilation tube as a highly promising alternative that could eliminate the need for secondary removal surgeries and reduce associated complications in human patients.

### 4.3. Correlation with In Vitro and In Vivo Data

The robust in vitro material characterization and processing optimization were instrumental in guiding the development of PLA prototypes with favorable in vivo performance. Stringent control during raw-material handling and injection molding ensured consistent tube production with desired properties and led to ed superior mechanical strength of the PLA tube. While collection of long-term in vitro degradation and mechanical strength data is ongoing, preliminary in vivo micro-CT imaging showed sustained presence and initial signs of absorption over 9 months, aligning with our 1–2-year target. Despite the observed decline in mechanical properties, the residual strength remains more than sufficient for the intended clinical application. Since the physiological force required to resist tympanic membrane retraction is minimal, the tube retains adequate load-bearing capacity for maintaining patency and preventing premature collapse. Maintained hearing function and minimal inflammatory response histologically underscore the successful translation of in vitro biocompatibility to the complex in vivo middle-ear environment.

Biological factors such as enzymatic activity, inflammatory pH fluctuations, and mucosal interactions can theoretically accelerate degradation compared to PBS. However, a critical countervailing factor is that the functional middle ear is an aerated cavity. Unlike the fully submerged in vitro model, the tube operates primarily in air, limiting water uptake. This condition may retard hydrolytic bulk erosion, potentially offsetting the accelerating biological effects.

Clinically, a 12–18-month ventilation period is optimal. Micro-CT confirmed tube integrity at 9 months. Given PLA’s bulk erosion kinetics, this structural preservation strongly suggests mechanical retention beyond the critical 12-month threshold, aligning with the desired clinical standard.

### 4.4. Future Directions and Clinical Translation

While the preclinical data are promising, further in vitro degradation and mechanical strength tests are essential to fully understand the long-term performance and absorption kinetics of the PLA tubes. Furthermore, future research will explore advanced functionalities, such as antibacterial coatings applied via dip-coating methods, to develop an “absorbable antibacterial middle-ear ventilation tube.” This enhancement could further mitigate the risk of postoperative infections. The next step involves initiating human clinical trials to evaluate the safety and efficacy of the PLA tubes, with the ultimate goal of improving patient outcomes and reducing the complications associated with current non-absorbable devices.

### 4.5. Study Limitations and Future Directions

Several limitations warrant mention. First, the surgical model involved implantation via the mastoid bulla rather than a clinical myringotomy, limiting our ability to assess functional ventilation and tympanic membrane healing. In future studies, we will utilize a trans-tympanic model to verify efficacy. Second, the small sample size (*n* = 5) was intended for preliminary safety screening; larger cohorts are needed for robust statistical conclusions regarding auditory function. Finally, we did not evaluate biofilm formation or fully simulate the complex physiological environment (e.g., enzymes and pH) of the human middle ear. Subsequent research will incorporate biofilm assays and physiologically relevant simulations to better predict clinical performance.

## 5. Conclusions

We successfully developed and preclinically evaluated a novel bioabsorbable PLA middle-ear ventilation tube. The prototype demonstrated dimensions comparable to existing commercial tubes and confirmed in vitro biocompatibility and was well-tolerated in a guinea pig model, without adverse reactions or impacts on hearing. Micro-CT imaging and histological analyses showed structural integrity and initial degradation over 9 months, suggesting compatibility with the desired degradation timeframe, although long-term variability requires further investigation. These findings indicate that the developed PLA tube is a promising alternative to non-absorbable tubes, potentially eliminating the need for secondary surgeries and reducing complications. Preclinical data support further clinical trials to validate its safety and efficacy in human patients.

## Figures and Tables

**Table 1:** Comparison Table of Middle Ear Ventilation Tube Products.

Material	Test Subjects	Degradation Time	Dimensions (ID/OD)	Biocompatibility	Manufacturing Method	Refs
Poly(bis(ethyl glycol)phosphazene) (PBE)	28 Hartley guinea pigs	53% degraded at 30 days, 25% functionality retained at 60 days	1.0 mm/ 1.8 mm	No infection or inflammatory response	N.A.	D’Eredità and Marsh [20]
50/50 Poly(D,L-lactide-co-glycolide) (PLGA-50) and Poly(L-lactic acid) (PLA)	20 Hartley guinea pigs	PLGA-50: 18.8 ± 8.1 days PLA: 63.2 ± 19.3 days	0.76 mm/1.36 mm	No infection, inflammation, or hearing loss	N.A.	Massey et al. [29]
Crosslinked glycosaminoglycan hydrogels (CMHA-SX, CSx) and polyesters (PLA, PLGA)	43 chinchillas	CMHA-SX, CSx: 7–8 days PLGA: 18.9 ± 6.4 days PLA: >30 days	1.27 mm/ 8 mm	CMHA-SX, CSx: High inflammation PLGA: Moderate PLA: Low	Hydrogel: centrifugal casting Polyester: silicone mold injection molding	Park et al. [21]
Gelatin with antibiotics and steroid suspension	14 adult patients	21–84 days (average 63 days)	N.A.	No obstruction or complications	N.A.	Skovlund et al. [35]
Polylactic acid (PLA) (70% D/30% L)	None (in vitro bacterial assay only)	N.A.	1.02 mm/ 2.7 mm	N.A.	Machined from solid PLA rods	Ludwick, et al. [34]
Poly(L-lactic acid) (PLA)	20 guinea pigs	>270 days	1.27 mm/ 3.0 mm & 1.02 mm/ 2.7 mm	No infection, inflammation, or hearing loss. Cytotoxicity and sensitization test passed.	Injection molding	This study

## Data Availability

The original contributions presented in the study are included in the article; further inquiries can be directed to the corresponding author.
